# Evaluation of cold resistance in pear (*Pyrus* L.) germplasms: integrating physiological and biochemical responses with anatomical traits under low temperature stress

**DOI:** 10.7717/peerj.21475

**Published:** 2026-06-29

**Authors:** Huan Liu, Jintao Xu, Minghui Ji, Longfei Li, Yue Yao, Lijuan Gao, Baofeng Hao

**Affiliations:** Changli Research Institute of Fruit, Hebei Academy of Agriculture and Forestry Sciences, Qinhuangdao, China

**Keywords:** *Pyrus* L., Cold resistance, Physiochemical responses, Anatomical structure

## Abstract

Low temperature stress severely restricts the cultivation and distribution of pear (*Pyrus* L.) germplasms, frequently resulting in frost injury and yield reduction. To accurately evaluate the cold resistance of pear germplasm resources, this study investigates the physiological and biochemical responses of one-year-old branches to different degrees of low-temperature stress, as well as differences in the tissue structure of these pear germplasms after low-temperature stress. In this study, 122 pear germplasms were classified into high (HR), medium (MR), and low (LR) cold-tolerance categories based on their semi-lethal temperature (LT_50_). Further analysis of pear germplasms with different levels of cold resistance revealed that, with decreasing temperature, HR germplasms exhibited smaller increases in relative electrolyte conductivity (REC) and malondialdehyde (MDA) content and higher accumulation of proline (Pro), soluble proteins (SP), soluble sugars (SS), and peroxidase activity compared with LR germplasms. In addition, the peak values of these indicators generally occurred at lower temperatures in HR germplasms. A correlation analysis and principal component analysis indicated that physiological indices, including REC, bound water/free water ratio, SS, and MDA, as well as branch anatomical traits related to xylem and cortex proportions, were closely associated with variation in LT_50_. An integrated assessment using membership function analysis produced rankings consistent with LT_50_-based clustering, supporting the reliability of the multivariate evaluation framework. Overall, this study establishes an integrated, indicator-based approach for evaluating cold resistance in pear germplasm by integrating physiological, biochemical, and anatomical characteristics. These results provide a theoretical basis and methodological reference for screening cold resistance germplasms.

## Introduction

Pear (*Pyrus* L.) is a significant economic fruit tree in temperate regions, extensively distributed in Asia, Africa, and Europe (Teng et al., 2015). China’s pear cultivation area and production rank among the top in the world, with Hebei Province being a major production region. Hebei leads the whole country in pear planting area, production, and export volume. However, the northern region of Hebei has harsh, cold winters with extremely low temperatures that often cause frost damage to pear trees, reducing pear yield. Therefore, evaluating the cold resistance of pear germplasms is essential for selecting and matching cold-resistant parent plants, thereby promoting the sustainable development of the pear industry in cold regions. Low temperature is a primary abiotic factor limiting the geographical distribution and growth of plants (Li et al., 2024). To resist cold stress, plants regulate their metabolic processes through morphological, physiological, and biochemical adaptations, thereby enhancing their cold resistance (Ritonga & Chen, 2020). When subjected to cold stress, plants initiate diverse defensive mechanisms, including membrane stabilization, osmotic regulation, and antioxidant enzyme systems (Dong et al., 2024). Under low temperature stress, the permeability of the plasma membrane can be further increased by lipid peroxidation of the cell membrane, resulting in electrolyte leakage (Zhang et al., 2023) and an elevation in relative electrical conductivity (REC). As a precise measure of plant cold resistance derived from the REC logistic equation, the low-temperature semi-lethal temperature (LT_50_) has wide application in cold resistance assessment (Karami et al., 2018; Sun et al., 2020). Malondialdehyde (MDA), the lipid peroxidation product of the membrane, increased significantly when exposed to cold stress, likely indicating the extent of impairment to the plant membrane system (Jalili et al., 2023; Niu et al., 2025; Velikova, Yordanov & Edreva, 2000).

Osmotic adjustment is another important plant defense mechanism against cold stress, mainly by maintaining cellular osmotic pressure, in addition to preventing low-temperature damage to plants by increasing the content of soluble proteins (SP), soluble sugars (SS), and proline (Pro) (Ghosh et al., 2022; Janmohammadi, Zolla & Rinalducci, 2015; Seydel et al., 2022). Low temperatures also induce excessive accumulation of reactive oxygen species (ROS) in plants, including superoxide anion, hydrogen peroxide, and hydroxyl radicals (Dong et al., 2024). Antioxidant enzymes like superoxide dismutase (SOD) and peroxidase (POD) play essential roles in maintaining ROS homeostasis, thus contributing to cold resistance. Consequently, the levels of antioxidant enzyme activity, including peroxidase (POD) and superoxide dismutase (SOD), serve as crucial indicators for evaluating plant cold resistance (Nath et al., 2016; Sheng et al., 2023). Water content distribution within plant tissues also influences cold resistance; a higher proportion of bound water relative to free water (BW/FW) reduces freezing point and enhances cold resistance (Wu, 2011).

Plant cold resistance is determined by both internal biochemical and physiological regulatory mechanisms as well as the anatomical configuration of branches (Buchner & Neuner, 2011). Xylem cells generally have rigid and thick cell walls, which provide mechanical protection and reduce freezing injury. In contrast, the cortex consists of living cells with thinner walls and lower dormancy (MacDonald et al., 2014).

Since a single indicator cannot reliably distinguish differences in cold resistance among germplasms, multidimensional evaluation systems have increasingly been used to assess cold resistance in fruit trees. For example, studies in apple have shown that integrating LT_50_ with multiple physiological indices can significantly improve the reliability of cold resistance evaluation (Jing et al., 2023). Integrated cold resistance assessment systems that combine physiological traits with anatomical characteristics have also been established in tree species such as raspberry, jujube, and peach (Chang et al., 2023; Li et al., 2024; Niu et al., 2023). These studies further indicate that multi-indicator evaluation approaches are effective and applicable for identifying cold-tolerant germplasm resources.

In contrast, existing studies on pear cold resistance have primarily focused on physiological and biochemical traits, and the research scope has often been limited to a single germplasm type or a small number of local cultivars (Guo et al., 2020; Qin et al., 2024; Wang et al., 2023). Systematic evaluations encompassing diverse pear germplasm types are rarely reported, and integrated, multi-level assessments integrating physiological and biochemical responses with branch anatomical characteristics are largely lacking in published literature.

Based on this background, the present study evaluated and classified the cold resistance of 122 pear germplasm resources representing diverse origins and types. Twelve representative germplasms with different cold resistance levels were further selected for a systematic analysis of their physiological, biochemical, and branch anatomical responses to low-temperature stress. Recognizing the inherent interspecific variation in cold resistance within *Pyrus*, we intentionally employed a multi-species panel (including *Pyrus ussuriensis*, *Pyrus bretschneideri*, *Pyrus pyrifolia*, and *Pyrus communis*) to broaden the phenotypic range of cold resistance (quantified by LT_50_) and to test whether key physio-biochemical and anatomical traits associated with cold resistance can be identified across species boundaries. Importantly, our goal was to establish an accession-level phenotypic evaluation framework based on such common markers, rather than to rank the innate cold resistance of different species. This study aimed to establish an integrated, multivariate evaluation system for pear cold resistance to provide theoretical support and methodological references for cold-resistant germplasm screening.

## Materials and Methods

### Schematic overview of the experimental program

**Figure 1:**
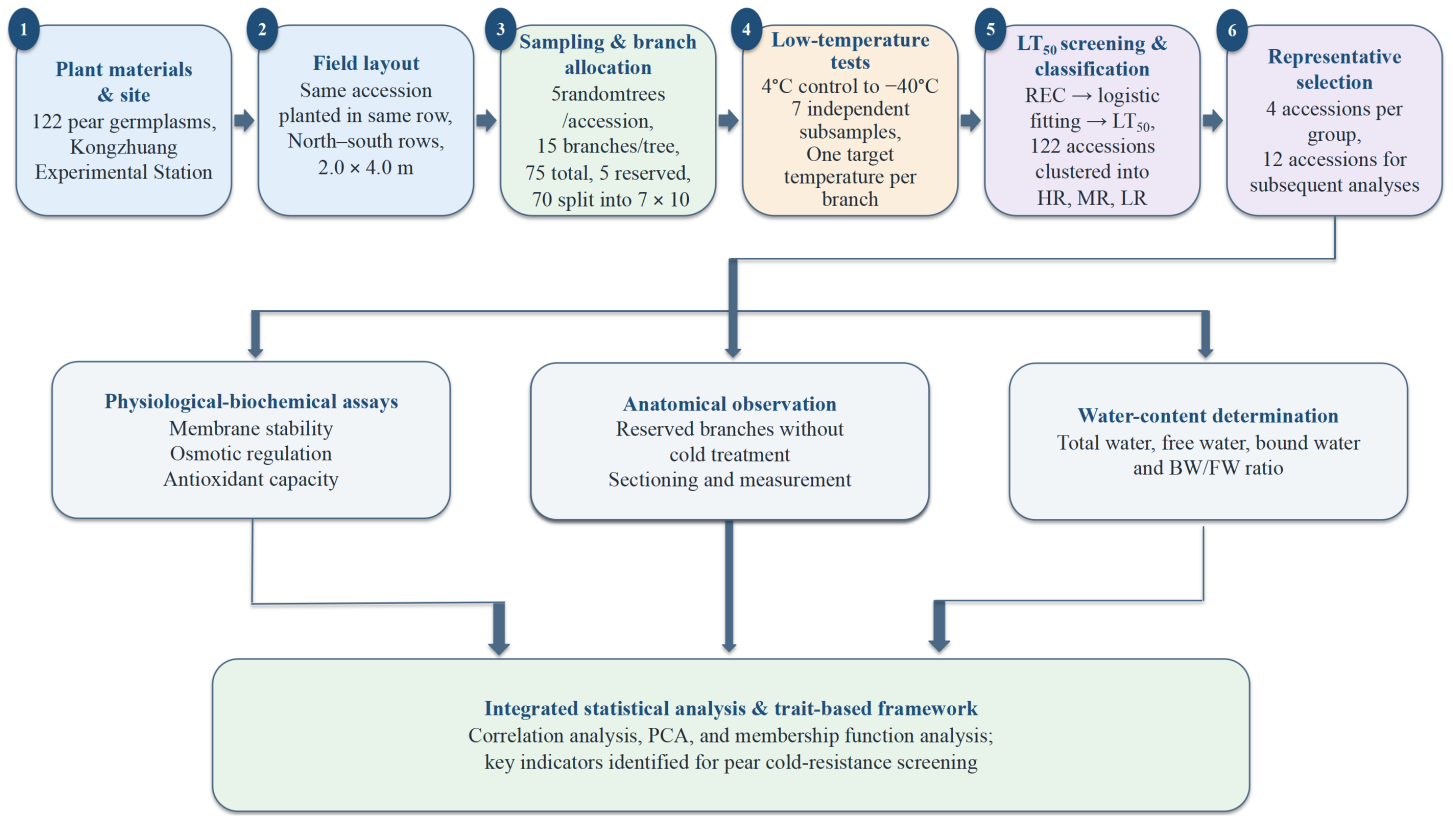
Schematic overview of the experimental program.

A schematic overview of the experimental program is shown in Fig. 1. The pear trees in the germplasm nursery were planted in north-south-oriented rows at a spacing of 2.0 m × 4.0 m, with trees of the same accession conventionally planted within the same row rather than arranged in a formal randomized complete block design. For each accession, five healthy, pest-free, similarly aged, and uniformly growing trees were randomly selected, and 15 annual branches were collected from each tree; of the 75 branches collected per accession, five were randomly reserved for anatomical observation and water-content determination, whereas the remaining 70 were evenly divided into seven independent sets for low-temperature treatments in a programmable high-low-temperature chamber. Relative electrolyte conductivity after the seven temperature treatments was used to estimate LT50 by logistic fitting, allowing the 122 pear germplasm accessions to be preliminarily classified into high-, medium-, and low-cold-resistance groups. Based on this classification, four representative accessions from each group were selected for subsequent physiological, biochemical, anatomical, and water-content analyses, and correlation analysis, principal component analysis, and membership function analysis were used to identify key indicators and establish a trait-based evaluation framework for screening cold-resistant pear germplasm. All experimental procedures followed the cited standard methods and conformed to good laboratory practices in the laboratory of the Changli Research Institute of Fruit, Hebei Academy of Agriculture and Forestry Sciences.

### Summary of the experimental field

**Figure 2:**
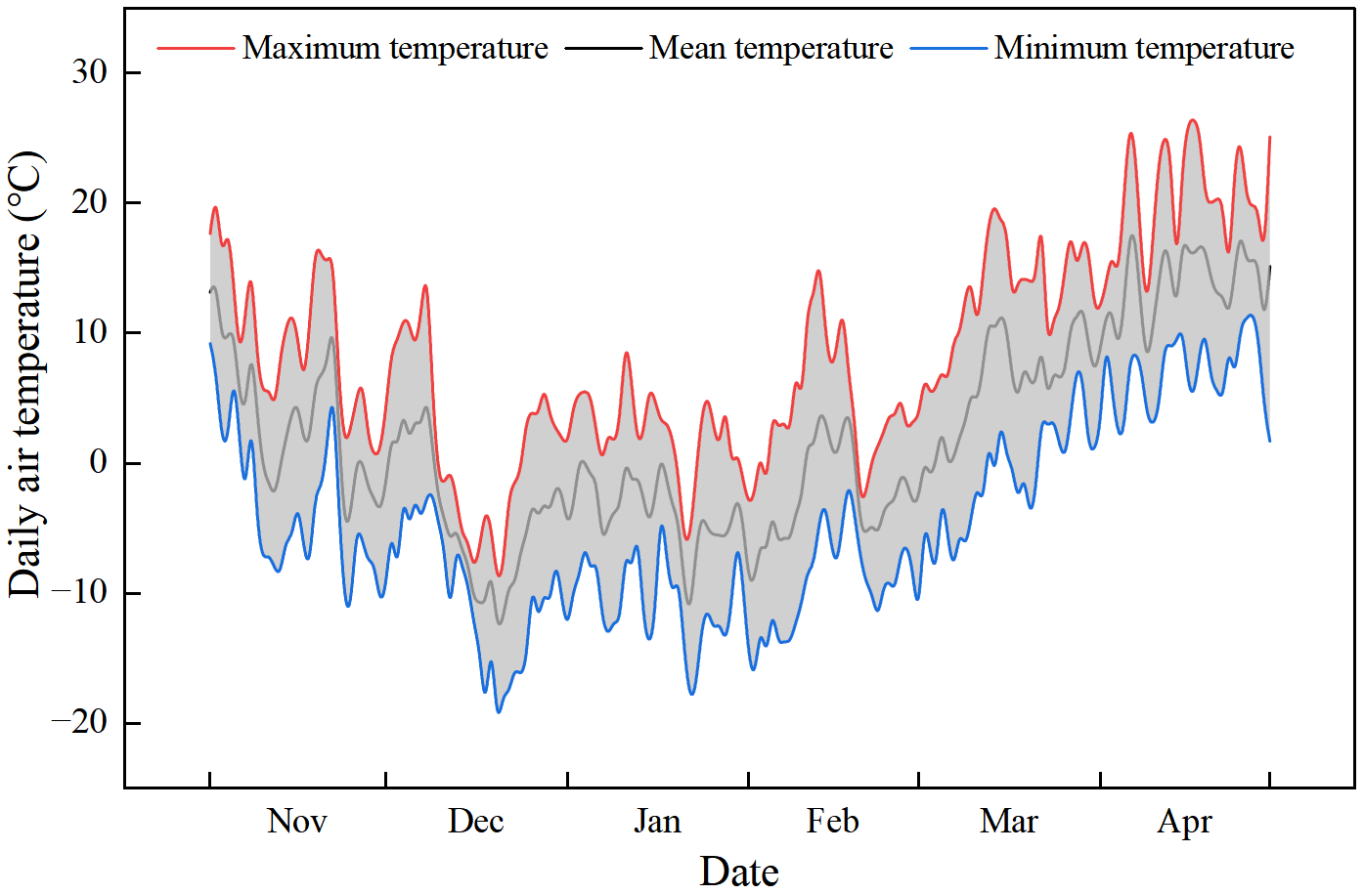
Daily mean air temperature at the experimental site from the overwintering period to the flowering period (November 2023 –April 2024).

The field experiment was conducted at the Kongzhuang Experimental Station, Changli Institute of Fruit Research, Hebei Academy of Agriculture and Forestry Sciences, China (39°42′N, 119°25′E). The site is located at an altitude of approximately 20 m and receives an average of 2,809 h of sunshine annually, with a mean annual temperature of 10 °C and an annual effective accumulated temperature of 3,940 °C at the ≥10 °C threshold. The frost-free period lasts about 186 days, and the average annual precipitation is approximately 640 mm. The experimental field is equipped with irrigation infrastructure to support routine orchard management. The region is characterized by a continental monsoon climate. During the overwintering period, meteorological data were collected from the experimental field. Figure 2 shows the daily average temperature records from November 2023 to April 2024. The site exhibited typical winter temperature fluctuations during this period, with temperatures gradually recovering to early spring levels after multiple cold events. These data provide environmental background information for the low-temperature conditions under which the pear germplasms overwintered and were evaluated.

### Plant materials

**Figure 3:**
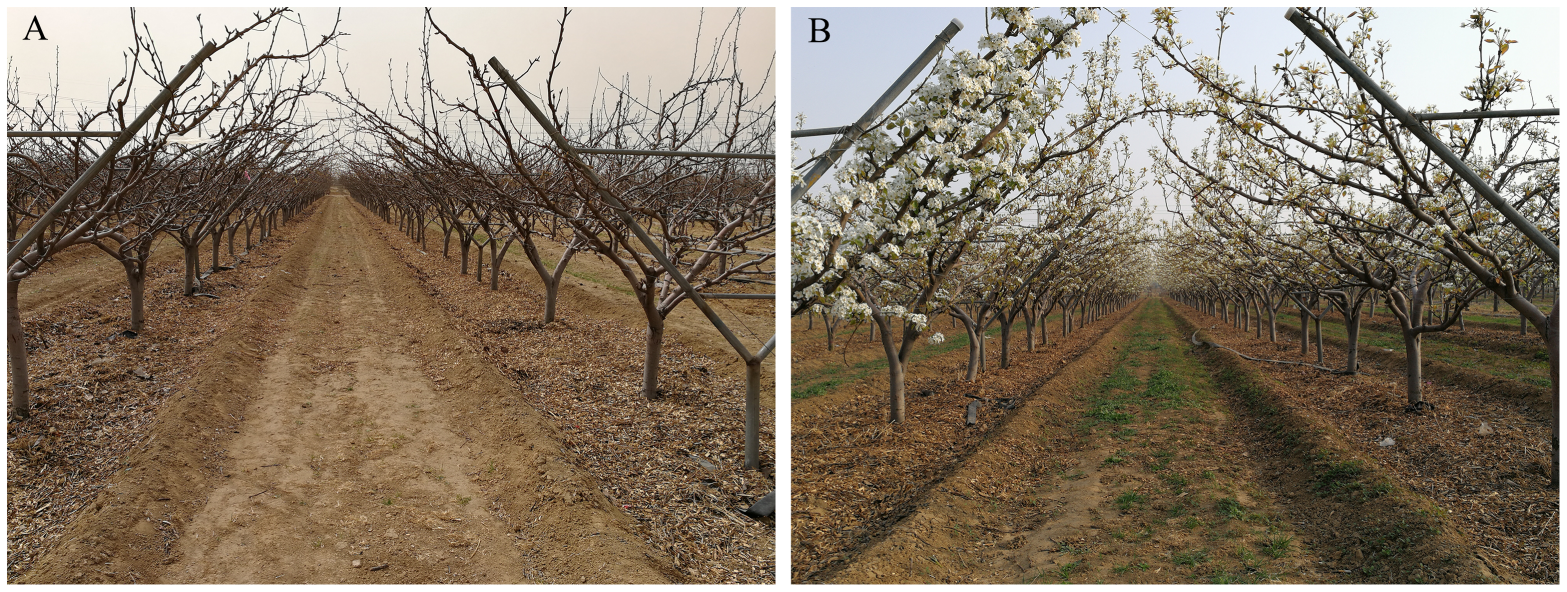
Growth status of tested materials. (A) Pictures of the dormant period (Image taken prior to sample collection), taken on 23 January 2024. (B) Images of the pear blossom period, taken on 17 April 2024.

A total of 122 pear germplasms were selected from the National Horticultural Crop Germplasm Resources Nursery in the Bohai Rim Region (Table S1). To reduce potential confounding effects and ensure comparability in cold resistance evaluations, all experimental trees were of the same age and rootstock: all were grafted in 2012 onto *Pyrus betulifolia* and were 12 years old at the time of the study. The trees were planted in a north-south orientation with a plant spacing of 2.0 m and a row spacing of 4.0 m. The germplasm accessions were maintained in an established orchard, with trees of the same accession conventionally planted within the same row rather than arranged in a formal randomized complete block design. Sampling was conducted on 22 January 2024 (Fig. 3). For each germplasm, five healthy, pest-free, and similarly aged trees exhibiting uniform growth were randomly selected from the available trees. From each tree, 15 annual branches were collected, each 40–50 cm in length and 0.8–1.0 cm in diameter. After being brought back to the laboratory, five branches were randomly selected from the 75 branches collected for each germplasm accession and used for anatomical observation and water-content determination. The remaining 70 branches were evenly divided into seven independent subsample sets, with 10 branches in each set, and subjected to low-temperature stress tests in the high–low-temperature test chamber (model: GDJS-500L) at seven temperature treatments: 4 °C (control), −15 °C, −20 °C, −25 °C, −30 °C, −35 °C, and −40 °C. Once the target temperature was reached, it was sustained for 12 h. Both the freezing and thawing rates were set to 4 °C h^−1^. Subsequently, the samples were placed in a refrigerator at 4 °C and thawed for 12 h before testing.

To systematically compare physiological responses and structural differences of pear germplasms at varying cold-tolerance levels under low-temperature stress, representative accessions were selected according to the within-category distribution of LT_50_. Twelve samples were selected following a unified principle: four germplasms were chosen from each level, ensuring that the selected materials maintained a certain degree of dispersion within the LT_50_ range of that level, rather than clustering near the extremes or central values of the interval. This sampling approach reduces the influence of any single accession and improves the robustness of subsequent multi-indicator comparisons. For physiological and biochemical analyses, flowers and leaf buds were removed, and the samples were immediately frozen in liquid nitrogen and stored at −80 °C for subsequent use. However, for the assessment of anatomical structures, fresh samples were used to exclude potential freezing artifacts. Each stress temperature and measurement parameter were repeated for three experiments.

### Experimental methods

#### Assessment of physio-biochemical indicators

Electrical conductivity was assessed using the methodology outlined by Wang et al. (2022). For each freezing temperature, three branches per variety were used and sliced into 1–2 mm segments. Subsequently, two g samples were weighed, and the leachate’s conductivity was determined using the DDS-307 conductivity meter (LeiCi, Sheng Ke, Shanghai, China).

The LT_50_, serving as the measure of frost hardiness, was determined from the inflection point of a logistic function fitted to the electrolyte leakage data. The relative conductivity (y) was fitted to a function of treatment temperature (t) using the equation: y = k/(1+ae^−bt^), where k indicates the saturation capacity of relative conductivity, and a and b are the function’s parameters (Lu, Hu & Li, 2017).

The pear germplasms with different cold resistance levels were preliminarily screened for the determination of oxidative stress indices, osmoregulatory substances, and antioxidant enzyme activities. MDA content was measured using the thiobarbituric acid reactive substances test (Hodges et al., 1999). The content of Pro was determined by the ninhydrin colorimetric method (Bates, Waldren & Teare, 1973). SP levels were evaluated with the G-250 Coomassie Brilliant Blue technique (Bradford, 1976). SS content levels were determined using the anthrone colorimetric method (Yemm & Willis, 1954). POD activity was quantified by the guaiacol method (Bradford, 1976). SOD activity was quantified using the nitro blue tetrazolium chloride method (Bradford, 1976).

The total water content (TW) of each branch was determined using the drying method (Chen et al., 2014). An Abbe refractometer was used to measure the free water (FW) amount (Chen et al., 2014). Bound water (BW) content was calculated as: BW = TW-FW (Chen et al., 2014).

#### Anatomical structure of branches

Three branches per pear variety were sampled, and sections were cut to a thickness of 10–12 µm using a cryostat (Leica CM1850). A SteREO Discovery V8 microscope (ZEISS, Oberkochen, Germany) was used for the observation and photographic recording of the sections, and each anatomical structure was also measured using the Digimi Zer 4.5.1 software. Three stems were then chosen from each sample, and each portion was viewed from three different angles for a total of nine observations.

\begin{eqnarray*}\mathrm{CR} \left( \text{cortex ratio} \right) & = \left( \text{Cortical thickness}/\text{Branch radius} \right) \times 100\% \end{eqnarray*}

\begin{eqnarray*}\mathrm{XR} \left( \text{xylem ratio} \right) & = \left( \text{Xylem thickness}/\text{Branch radius} \right) \times 100\% \end{eqnarray*}

\begin{eqnarray*}\mathrm{PR}1 \left( \text{phloem ratio} \right) & = \left( \text{Phloem thickness}/\text{Branch radius} \right) \times 100\% \end{eqnarray*}

\begin{eqnarray*}\mathrm{PR}2 \left( \text{pith ratio} \right) & = \left( \text{Pith thickness}/\text{Branch radius} \right) \times 100\% \end{eqnarray*}

\begin{eqnarray*}\mathrm{X}/\mathrm{C} \left( \mathrm{xylem}/\text{cortex thickness} \right) & =\text{Thickness of xylem}/\text{Thickness of cortex} \end{eqnarray*}

### Statistical analysis

Microsoft Excel 2011 software and SPSS Statistics 24 software (IBM Corp., USA) were used for all statistical analyses. Cluster analysis, principal component analysis (PCA), and correlation analysis were conducted using Origin 2021 (OriginLab Corp., Northampton, MA, USA). All data are presented as the mean ±  standard deviation (SD) of three biological replicates. Before statistical analysis, homogeneity of variances was tested. Differences between groups were examined using one-way analysis of variance (ANOVA) followed by Duncan’s multiple range test, with the significance level set at *P* < 0.05. The cluster analysis was based on Euclidean distance. Correlation analyses used Pearson’s correlation coefficient, and significance levels were set at *P* < 0.05 and *P* < 0.01. Principal component analysis employed standardized variables (*z*-scores). Cold resistance was evaluated using the values of the affiliation function (Xiu et al., 2019), based on the following formulas:

For indices exhibiting a positive association with cold resistance:

\begin{eqnarray*}\mathrm{U} \left( {\mathrm{X}}_{\mathrm{ij}} \right) = \left( {\mathrm{X}}_{\mathrm{i}}-{\mathrm{X}}_{\mathrm{min}} \right) / \left( {\mathrm{X}}_{\mathrm{max}}-{\mathrm{X}}_{\mathrm{min}} \right) \end{eqnarray*}

For indices exhibiting a negative association with cold resistance:

\begin{eqnarray*}\mathrm{U} \left( {\mathrm{X}}_{\mathrm{ij}} \right) =1- \left[ \left( {\mathrm{X}}_{\mathrm{i}}-{\mathrm{X}}_{\mathrm{min}} \right) / \left( {\mathrm{X}}_{\mathrm{max}}-{\mathrm{X}}_{\mathrm{min}} \right) \right] \end{eqnarray*}

X_i_ is the observed value of the index for the i-th sample, and X_min_ represents the minimum value of this indicator across all samples, while X_max_ represents the maximum value.

## Results

### Cluster analysis of cold resistance in pear germplasms

**Figure 4:**
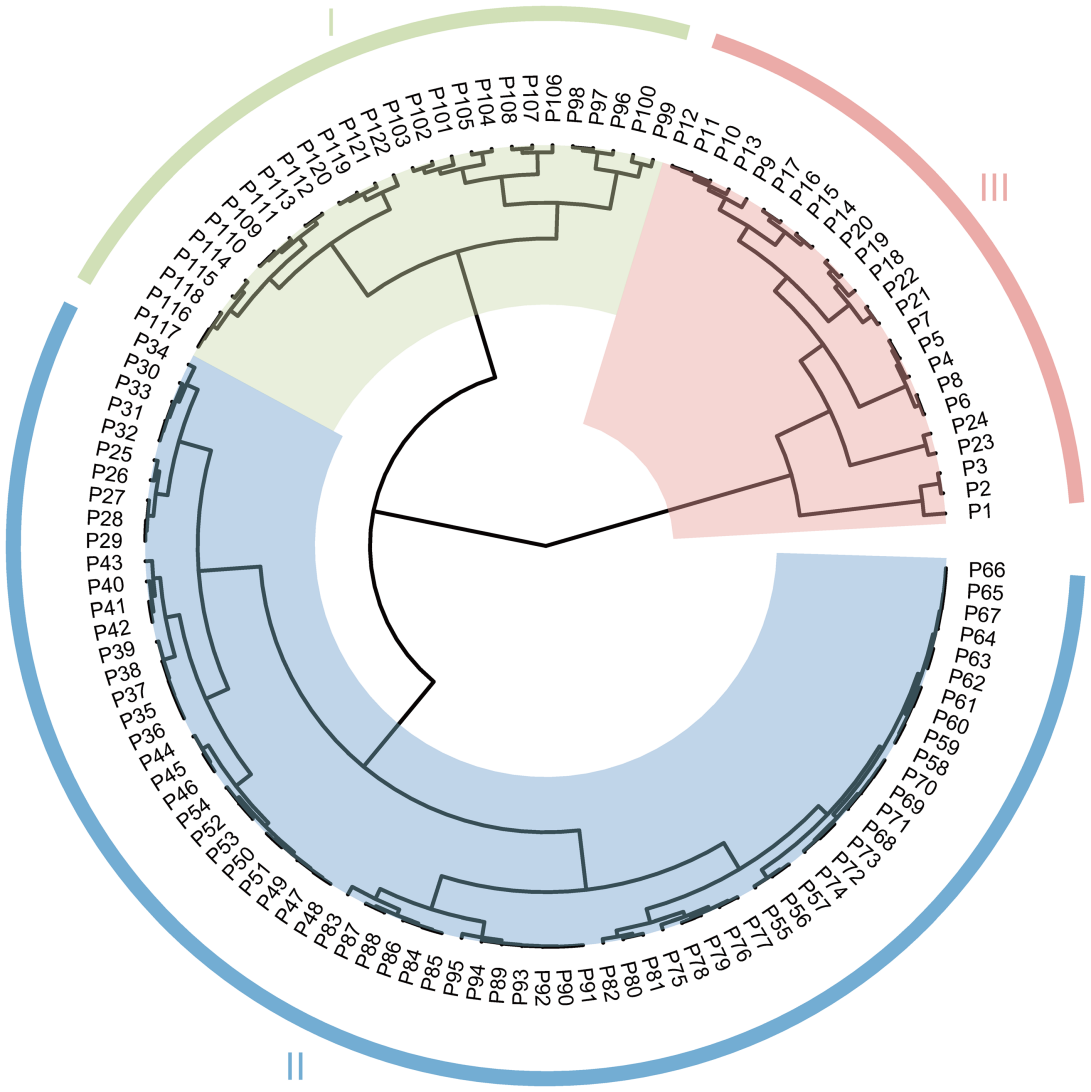
Cluster analyses of 122 pear germplasms according to the LT_50_. Hierarchical cluster analysis of 122 pear germplasms based on LT_50_, performed using Ward’s method with Euclidean distance as the distance metric.

Temperature was designated as the independent variable (x), while REC measured in branch samples served as the dependent variable (y). Each pear germplasm’s cold stress response curves were fitted using a logistic regression analysis. LT_50_ under low temperature was calculated accordingly. Based on the LT_50_ values of 122 germplasms, a cluster analysis (Fig. 4) identified three distinct groups. Cluster I represents the low cold-resistant group (LR), comprising 27 germplasms, primarily *P. pyrifolia* and *P. communis*. Cluster II indicates the medium cold-resistant group (MR), which includes 71 germplasms, mainly *P. bretschneideri* and *P. pyrifolia*. Cluster III indicates the high cold-resistant group (HR), consisting of 24 germplasms, predominantly *P. ussuriensis*.

The LT_50_ values were distinctly separated among the cold-tolerance groups: −35.68 to −32.17 °C for the HR group, −31.42 to −28.65 °C for the MR group, and −28.24 to −25.98 °C for the LR group (Table S2). The coefficients of variation within the HR, MR, and LR groups were low (2.54%, 2.52%, and 2.51%), indicating minimal phenotypic variation in LT_50_ within each group. Given the significant differences between the groups and the relatively small variations within each group, four representative samples were selected from the HR, MR, and LR groups for subsequent physiological, biochemical, and anatomical analyses.

### Responses of cell membrane stability to low temperatures

**Figure 5:**
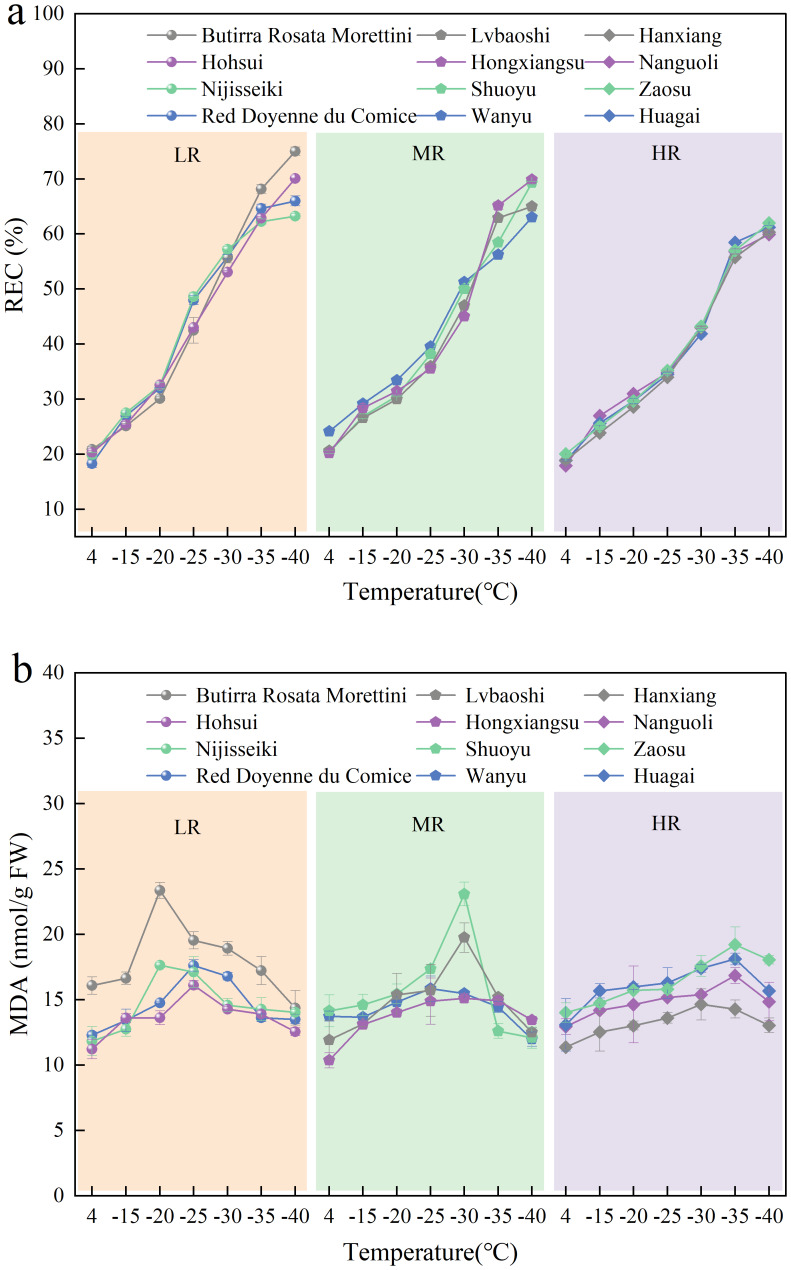
Relative conductance (REC) and Malondialdehyde (MDA) content changes in pear tree branches subjected to cold stress. (A) REC values in branch samples of 12 pear varieties. (B) MDA levels in branch samples of 12 pear cultivars. Data are presented as the mean ± standard deviation (SD) of three biological replicates. Differences among temperature treatments within each cultivar were analyzed by one-way ANOVA followed by Duncan’s multiple range test, with statistical significance set at *p* < 0.05. The error bars represent the standard errors.

The REC of branches in 12 pear germplasms showed a gradually increasing trend (Fig. 5A), and the overall change followed an S-shaped curve, with the inflection point ranging from −20 °C to −25 °C. However, the rate of increase in REC varied among germplasms with different levels of cold resistance. Germplasms in MR and HR showed a gradual increase in REC from 4 °C to −25 °C, followed by a steep rise from −25 °C to −35 °C. In contrast, LR germplasms had a significant increase in REC starting at −20 °C. Additionally, at extremely low temperatures, RECs differed significantly among germplasm types, with REC following a LR > MR > HR trend.

As shown in Table 1, the correlation coefficients R^2^ of the fitted logistic equations for the 12 germplasms ranged from 0.84 to 0.92. The LT_50_ values ranged from −26.58 °C to −35.16 °C. Among the HR germplasms, ‘Hanxiang’ had the lowest LT_50_, whereas ‘Butirra Rosata Morettini’ had the highest LT_50_ among the LR germplasms.

**Table 1:** Logistics equations and low-temperature half-lethal temperatures (LT_50_) for twelve distinct pear germplasms.

Germplasm	Logistics equation	LT_50_ (°C)	R^2^
Nanguoli	y = 100/(1 + 4.71e^−0.0453t^)	−34.19	0.92
Huagai	y = 100/(1 + 4.75e^−0.0456t^)	−34.17	0.88
Hanxiang	y = 100/(1 + 4.86e^−0.0450t^)	−35.16	0.88
Zaosu	y = 100/(1 + 4.56e^−0.0445t^)	−34.10	0.87
Wanyu	y = 100/(1 + 3.50e^−0.0401t^)	−31.25	0.88
Shuoyu	y = 100/(1 + 4.52e^−0.0499t^)	−30.21	0.88
Lvbaoshi	y = 100/(1 + 4.43e^−0.0483t^)	−30.82	0.87
Hongxiangsu	y = 100/(1 + 4.59e^−0.0513t^)	−29.72	0.84
Hosui	y = 100/(1 + 4.52e^−0.0534t^)	−28.24	0.89
Butirra Rosata Morettini	y = 100/(1 + 4.81e^−0.0591t^)	−26.58	0.85
Red Doyenne du Comice	y = 100/(1 + 4.59e^−0.0549t^)	−27.75	0.92
Nijisseiki	y = 100/(1 + 4.12e^−0.0503t^)	−28.18	0.91

**Notes.**

LT_50_ was estimated as the inflection point of the logistic model fitted to REC-temperature data for each cultivar (*n* = 3). R^2^ indicates goodness of fit.

As the temperature decreased, MDA content showed an increase–decrease pattern in all tested germplasms (Fig. 5B). MDA content was higher under low-temperature treatments than under the control in all 12 germplasms. In most HR and MR germplasms, MDA content peaked at −30 °C to −35 °C, except for ‘Wanyu’. In the LR germplasms, MDA content peaked at −20 °C to −25 °C. Compared with the LR germplasms, the HR germplasms showed smaller increases in MDA content across the temperature treatments.

### Changes in osmotic regulators in response to low-temperature conditions

**Figure 6:**
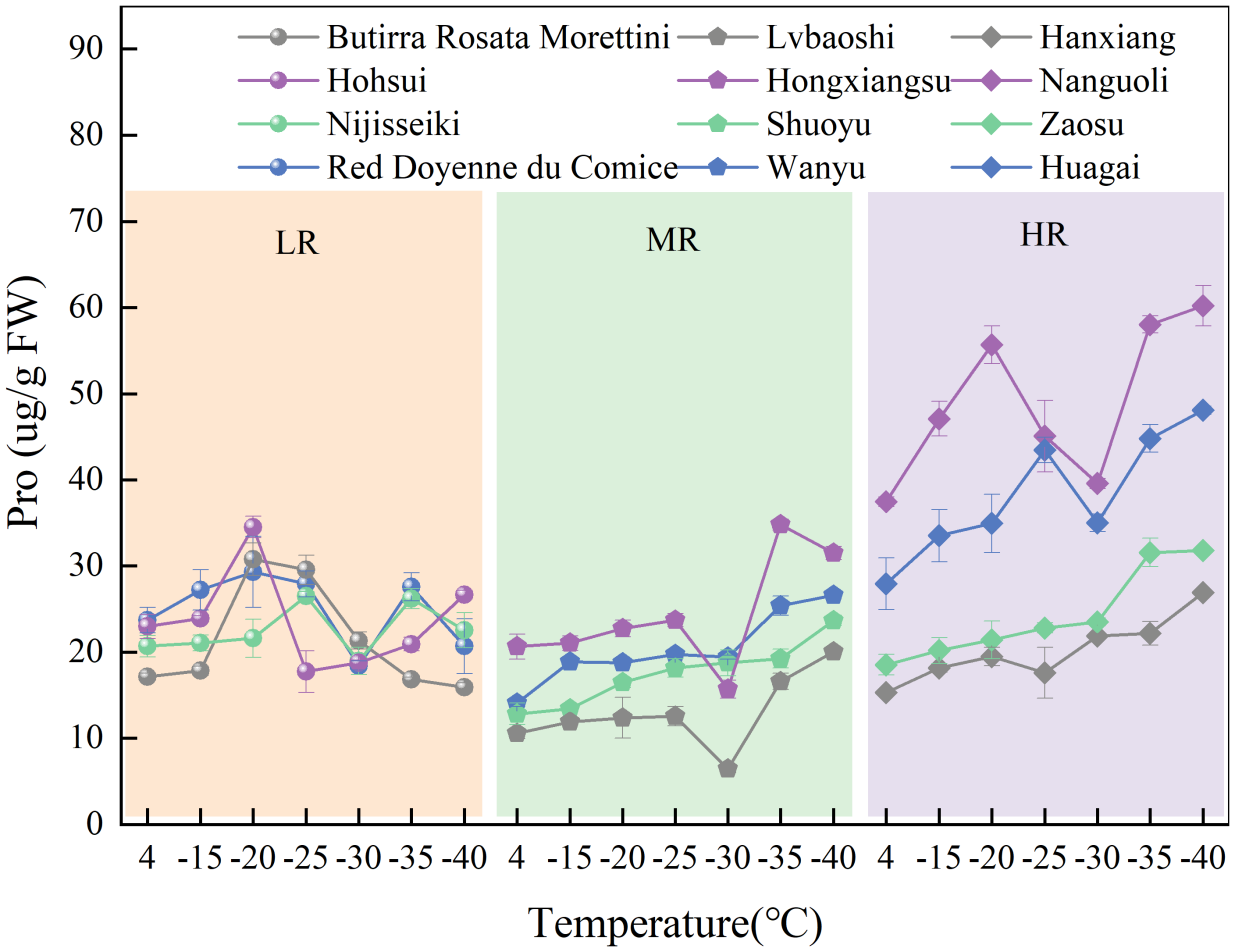
Proline (Pro) content changes in 12 pear varieties under different low-temperature stresses ranging from 4 °C to −40 °C. Data are presented as the mean ± standard deviation (SD) of three biological replicates. Differences among temperature treatments within each cultivar were analyzed by one-way ANOVA followed by Duncan’s multiple range test, with statistical significance set at *p* < 0.05. The error bars represent the standard errors.

The Pro accumulation levels of HR and MR germplasms generally increased with decreasing temperature (Fig. 6), with most reaching a peak at −40 °C, and there was a significant difference (*p* < 0.05) between the Pro peak levels and the control groups, with increases ranging from 60.7% to 89.6%. Notably, HR germplasms ‘Nanguoli’ and ‘Huagai’ maintained relatively high levels of Pro content and consistently exhibited higher Pro content than other varieties. In contrast, the Pro content in LR germplasms initially increased and then declined, reaching a peak between −20 °C and −25 °C. The increase in Pro content in LR germplasms ranged from 23.6% to 79.5%.

**Figure 7:**
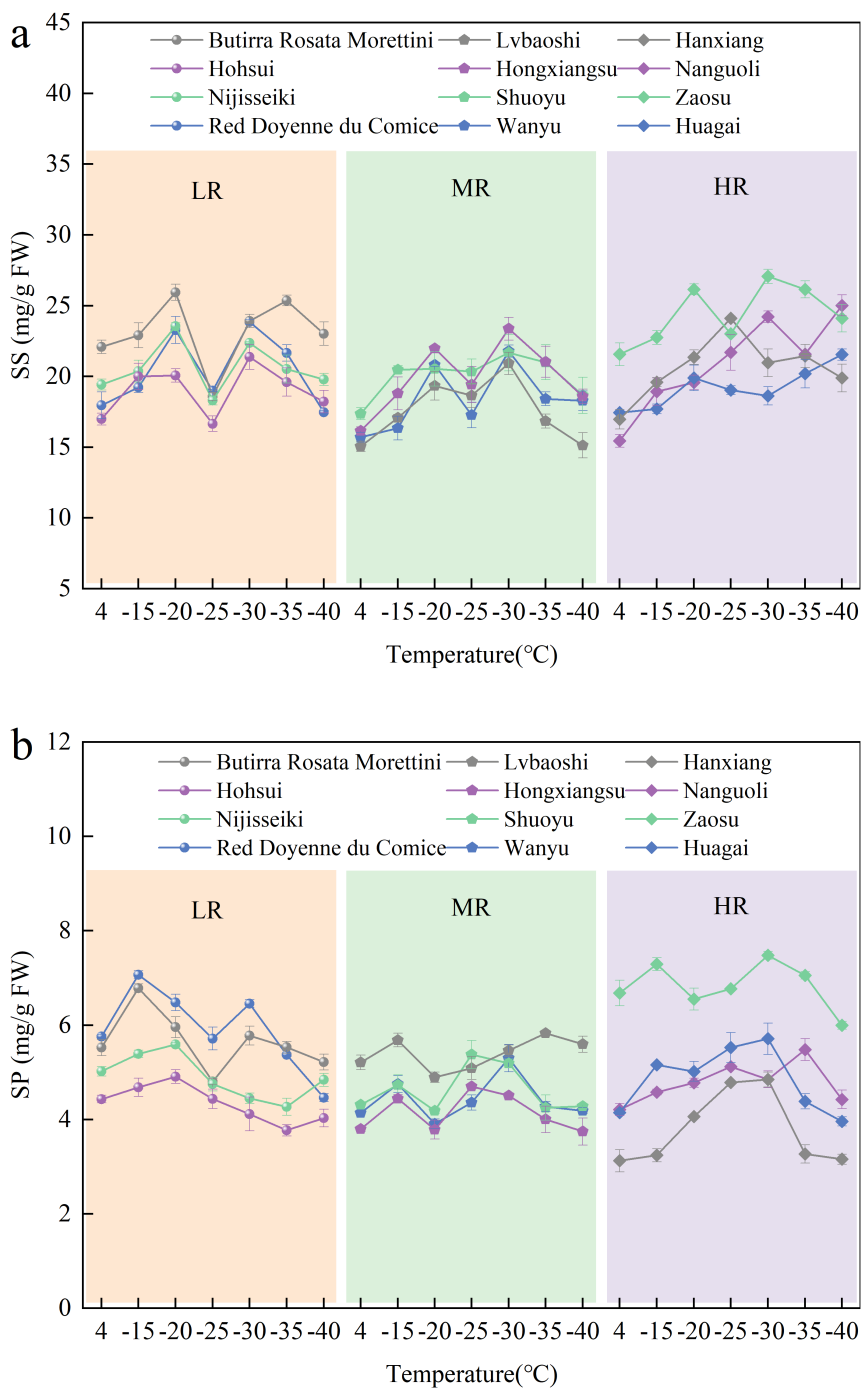
Soluble sugar (SS) and soluble protein (SP) content changes in pear tree branches under different low-temperature stresses. (A) SS concentrations in branch samples of 12 pear varieties. (B) SP concentrations in branch samples of 12 pear varieties. Data are presented as the mean ± standard deviation (SD) of three biological replicates. Differences among temperature treatments within each cultivar were analyzed by one-way ANOVA followed by Duncan’s multiple range test, with statistical significance set at *p* < 0.05. The error bars represent the standard errors.

Under low-temperature stress, the contents of soluble proteins (SP) and soluble sugars (SS) in pear branches exhibited alternating increase–decrease–increase–decrease patterns (Fig. 7). Both SS and SP content in HR germplasms reached their peak values at −30 °C and below. In contrast, LR germplasms exhibited maximum SS content at −20 °C and −30 °C, while the highest SP concentrations occurred at −15 °C and −20 °C. These results indicate that, under low-temperature stress, the accumulation of osmotic adjustment substances in LR germplasms tends to occur at relatively higher temperatures compared with that in HR germplasms.

The increase in SS content under cold stress was greater in HR germplasms (23.5%–61.9%) than in LR ones (17.5%–32.7%). Similarly, the increase in SP content was also higher in HR germplasms (12.0%–55.4%) than in LR germplasms (10.8%–22.8%).

**Figure 8:**
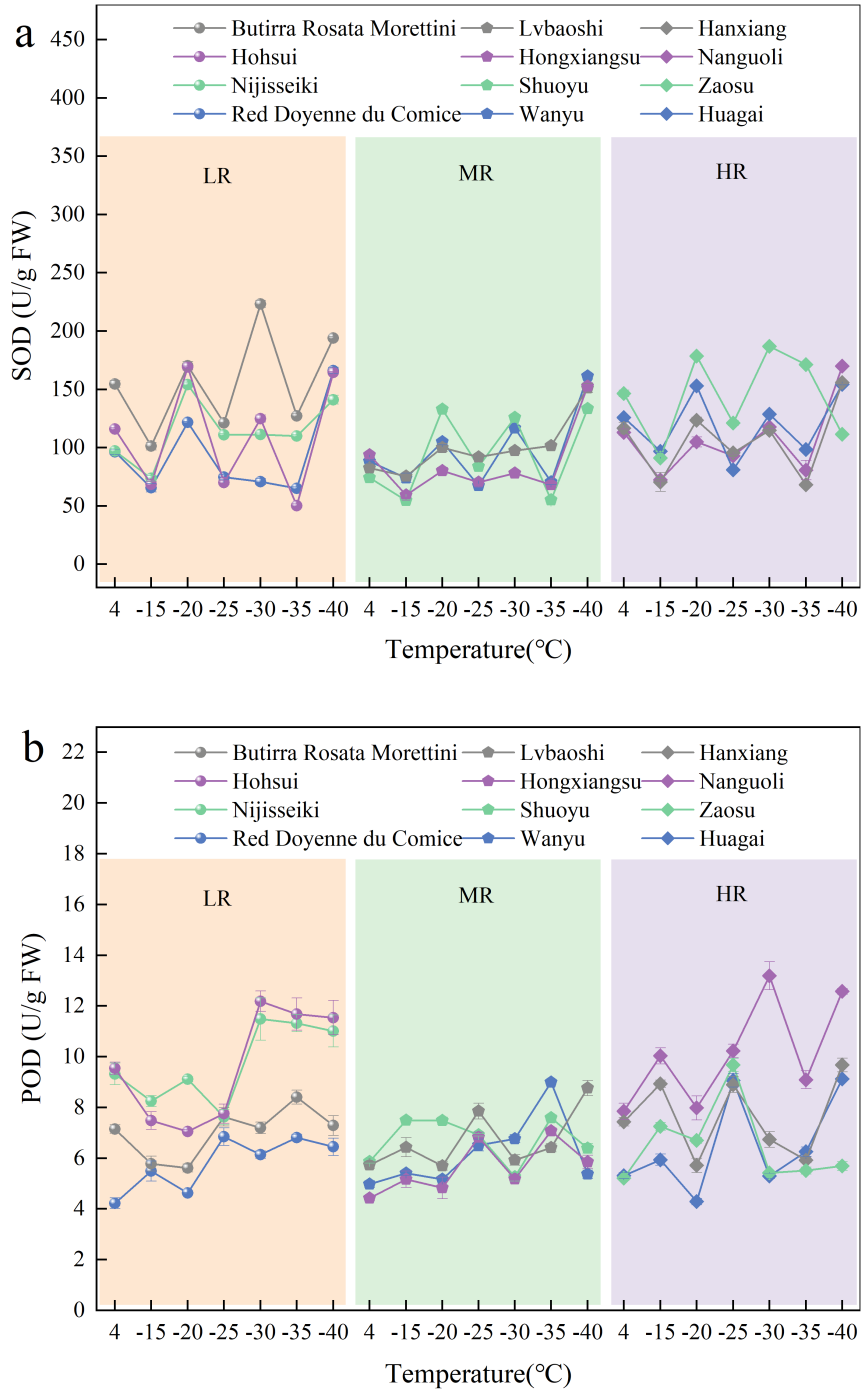
Superoxide dismutase (SOD) and Peroxidase (POD) activity changes in pear tree branches under different low-temperature stresses. (A) SOD activity levels quantified across 12 pear cultivars. (B) POD activity levels quantified across 12 pear cultivars. Data are presented as the mean ± standard deviation (SD) of three biological replicates. Differences among temperature treatments within each cultivar were analyzed by one-way ANOVA followed by Duncan’s multiple range test, with statistical significance set at *p* < 0.05. The error bars represent the standard errors.

### Changes in antioxidant enzyme activity in response to low- temperature conditions

Activity levels of SOD and POD in pear germplasm branches exhibited overall increasing trends during low-temperature stress, although fluctuations were observed across different temperatures and germplasms (Fig. 8). In HR and MR germplasms, SOD activity peaked at −40 °C at significantly higher levels than control levels (*p* < 0.05), with increases ranging from 22.8% to 82.1%. In the LR germplasms, such as ‘Nijisseiki’ and ‘Hosui’, reached their maximum SOD activity at −20 °C, with increases of 58.1% and 41.5%, respectively. In terms of POD activity, the majority of HR and MR germplasms demonstrated enzyme activity peaking at lower temperatures (−30 °C to −40 °C) than in LR germplasms (−25 °C to −35 °C). The increases in SOD activity in the LR germplasms ‘Butirra Rosata Morettini’, ‘Nijisseiki’, and ‘Hosui’ were 30.0%, 17.0%, and 27.8%, respectively.

### Branch water content analysis of annual branches

Table 2 shows significant differences in BW/FW ratios among the tested pear germplasms. HR germplasms exhibited the highest BW/FW ratios, ranging from 0.11 to 0.13, among which ‘Nangguo Pear’ had the highest ratio of 0.13. MR germplasms had BW/FW ratios ranging from 0.06 to 0.09. In contrast, LR germplasms exhibited the lowest ratios, ranging from 0.04 to 0.05, with ‘Butirra Rosata Morettini’ displaying the lowest ratio at 0.04. Overall, the BW/FW ratio followed the order HR >MR >LR, and the difference between HR and LR germplasms was significant (*p* < 0.05).

### Anatomical structure analysis of branches

The pear branch’s anatomical structure from inside to outside is pith, xylem, phloem, and cortex. Measurements of the anatomical structure of the branches of 12 different pear germplasms revealed substantial variation in thickness and proportion of these tissues among cultivars. To eliminate interference from inherent size differences among branches, this study selected proportional indicators of branch structures for analysis. All observations were consistently conducted on one-year-old branches. As shown in Table 3, the XR and X/C ratios were higher in HR germplasms than in LR germplasms, with significant differences between the two groups (*p* < 0.05). The CR and PR1 were lower in HR germplasms than in LR germplasms. Among the tested germplasms, ‘Hanxiang’ had the highest XR and X/C ratios and the lowest CR and PR1, whereas ‘Hosui’ had the lowest X/C ratio and the highest CR value.

### Integrated evaluation of cold adaptation capacity across pear cultivars

#### Correlation analysis of cold resistance index of pear

**Figure 9:**
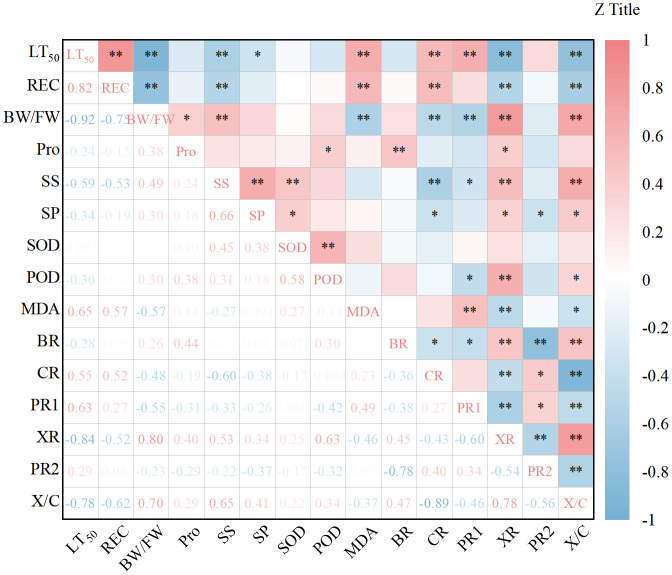
Pearson’s correlation analysis was conducted between LT_50_ and 14 cold resistance indexes of pear, including physiological, biochemical, and branch anatomical features. Two asterisks (**) indicate an exceptionally significant correlation (*p* < 0.01); one asterisk (*) denotes a statistically evident relationship (*p* < 0.05).

LT_50_ showed a correlation with both physiological indicators and anatomical structures of pear annual branches. As shown in Fig. 9, LT_50_ showed a strong positive correlation with REC, MDA, CR, and PR1 (*p* < 0.01). LT_50_ showed highly significant negative correlations with BW/FW, SS, XT, X/C, and XR at *p* < 0.01 and with SP at *p* < 0.05, with the strongest correlation being with BW/FW (*r* = −0.92).

Among the 14 cold-resistance-related indicators, nine indices were significantly correlated with LT_50_. Physiological indicators also showed correlations with anatomical traits. SS and SP were significantly negatively correlated with CR and positively correlated with XR and X/C, whereas SOD showed no significant correlation with any anatomical trait.

#### Principal component analysis

**Figure 10:**
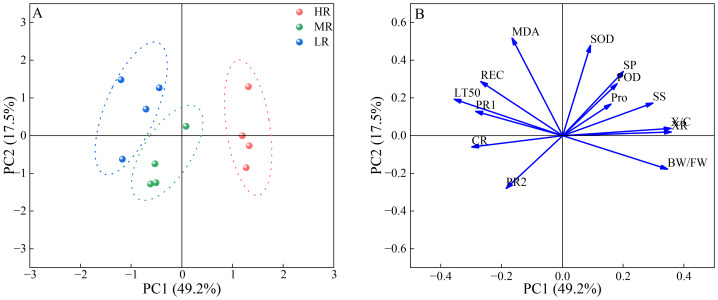
Principal component analysis was performed on *z*-score of 14 cold-resistance indices across 12 pear cultivars. (A) The segregation of 12 pear varieties over distinct quadrants under low temperature stress. (B) PCA biplot demonstrates the connection of 14 cold-resistance indices.

Principal component analysis (PCA) was conducted using LT_50_values alongside 13 additional parameters associated with cold resistance. PC1 and PC2 explained 49.2% and 17.5% of the total variance, respectively, with a cumulative contribution of 66.7% (Figs. 10A, 10B). As shown in Fig. 10A, 14 trait indices effectively divided pear germplasms into different cold-resistance groups across the four quadrants of the PCA plot. LR varieties were mainly distributed in Quadrant I, MR varieties were mainly located in Quadrant IV and near the origin, while HR varieties were distributed in Quadrants I and II. HR and LR germplasms were separated along PC1. Based on the loading plot and score plot (Figs. 10A, 10B), LT_50_, REC, MDA, PR1, and CR were positively correlated with LR varieties, while XR, X/C, BW/FW, SS, and Pro were positively correlated with HR varieties.

**Table 2:** Test results of moisture index of branches from 12 pear varieties.

**Variety**	**TW(%)**	**FW(%)**	**BW(%)**	**BW/FW**
Nanguoli	49.51 ± 0.26b	43.86 ± 0.57c	5.65 ± 0.59a	0.13 ± 0.02a
Huagai	51.49 ± 0.27a	46.38 ± 0.15b	5.12 ± 0.42ab	0.11 ± 0.01b
Hanxiang	49.16 ± 0.45bc	44.44 ± 0.48c	4.72 ± 0.5bc	0.11 ± 0.01b
Zaosu	45.19 ± 0.27e	40.83 ± 0.51d	4.36 ± 0.34c	0.11 ± 0.01b
Wanyu	48.18 ± 0.32cd	44.14 ± 1.11c	4.04 ± 0.79c	0.09 ± 0.02b
Shuoyu	47.57 ± 0.37d	44.46 ± 0.58c	3.11 ± 0.22de	0.07 ± 0.01cd
Lvbaoshi	47.92 ± 1.78d	44.65 ± 1.92d	3.27 ± 0.15d	0.07 ± 0.01c
Hongxiangsu	47.6 ± 0.56d	44.81 ± 0.94d	2.79 ± 0.39def	0.06 ± 0.01cde
Hosui	51.05 ± 0.66a	48.69 ± 0.56a	2.37 ± 0.12ef	0.05 ± 0ef
Butirra Rosata Morettini	51.45 ± 0.55a	49.32 ± 0.62a	2.13 ± 0.21f	0.04 ± 0f
Red Doyenne du Comice	51.32 ± 0.63a	48.81 ± 0.78a	2.51 ± 0.15def	0.05 ± 0def
Nijisseiki	51.24 ± 0.44a	48.58 ± 0.75a	2.66 ± 0.44def	0.05 ± 0.01c–f

**Notes.**

Data are presented as the mean ± standard deviation (SD) of three biological replicates. Different letters indicate significant differences among cultivars based on one-way ANOVA followed by Duncan’s multiple range test (*p* < 0.05).

**Table 3:** Index of anatomical structure of pear branches.

**Variety**	**Radius of branch (μm)**	**Thickness of cortex (μm)**	**Thickness of phloem (μm)**	**Thickness of xylem (μm)**	**Thickness of pith (μm)**	**The ratio of cortex (%)**	**The ratio of phloem (%)**	**The ratio of xylem (%)**	**The ratio of pith (%)**	**The xylem-cortex ratio**
Nanguoli	3,086.05 ± 137.85cd	188.20 ± 8.34bcd	283.10 ± 40.94de	2,029.69 ± 77.58c	780.35 ± 26.41ab	6.11 ± 0.49bcd	9.20 ± 1.55c	65.79 ± 1.17a	25.31 ± 1.17b	10.8 ± 0.73a
Huagai	3,701.80 ± 112.07a	212.88 ± 25.15abc	348.95 ± 42.95a–d	2,429.60 ± 129.13a	662.77 ± 41.83cde	5.74 ± 0.51cd	9.41 ± 0.88c	65.70 ± 4.5a	17.89 ± 0.59e	11.54 ± 1.63a
Hanxiang	3,399.54 ± 169.33b	190.50 ± 19.5bcd	316.60 ± 27.39cde	2,256.35 ± 96.69b	632.50 ± 67.08de	5.60 ± 0.33d	9.35 ± 1.23c	66.42 ± 2.8a	18.58 ± 1.27de	11.92 ± 1.24a
Zaosu	2,602.66 ± 62.22fg	156.10 ± 13.67d	274.88 ± 31.06e	1,723.50 ± 65.22d	535.45 ± 33.36f	6.00 ± 0.6bcd	10.57 ± 1.31bc	66.23 ± 2.26a	20.6 ± 1.75cde	11.08 ± 0.75a
Wanyu	3,069.47 ± 127.58cd	226.79 ± 21.85ab	346.18 ± 46.64a–d	1,735.36 ± 65.83d	771.75 ± 43.74ab	7.42 ± 1.03ab	11.25 ± 1.05bc	56.54 ± 0.47bc	25.14 ± 0.92b	7.71 ± 0.99b
Shuoyu	3,117.48 ± 114.2d	156.62 ± 12.11d	390.34 ± 36.8ab	1,730.95 ± 62.92d	712.80 ± 62.45a–d	5.03 ± 0.5d	12.5 ± 0.73ab	55.53 ± 0.51cd	22.92 ± 2.62bc	11.11 ± 1.2a
Lvbaoshi	2,465.39 ± 113.77gh	179.36 ± 18.15cd	263.96 ± 28.92e	1,296.98 ± 67.4gh	785.67 ± 49.13a	7.3 ± 1.01ab	10.76 ± 1.61bc	52.6 ± 0.55cde	31.89 ± 2.09a	7.30 ± 1.09b
Hongxiangsu	2,311.94 ± 155.43 h	169.07 ± 17.59d	275.96 ± 54.84e	1,220.03 ± 95 h	724.62 ± 26.75abc	7.3 ± 0.27ab	11.87 ± 1.61b	52.75 ± 1.07cde	31.38 ± 0.98a	7.23 ± 0.26b
Hosui	2,862.10 ± 88.87de	229.29 ± 31.1ab	358.30 ± 28.71abc	1,527.02 ± 106.49ef	707.02 ± 41.56a–d	7.99 ± 0.84a	12.51 ± 0.66ab	53.31 ± 2.24cde	24.69 ± 0.79b	6.71 ± 0.59b
Butirra Rosata Morettini	2,813.94 ± 168.09ef	198.81 ± 19.89bcd	398.42 ± 21.99a	1,386.76 ± 60.79fg	713.91 ± 19.05a–d	7.11 ± 1.13abc	14.18 ± 0.86a	49.31 ± 0.9e	25.43 ± 1.63b	7.04 ± 0.94b
Red Doyenne du Comice	3,157.22 ± 146.91c	226.15 ± 23.97ab	327.3 ± 16.43bcde	1,644.53 ± 88.39de	609.53 ± 40.96ef	7.16 ± 0.67abc	10.39 ± 0.91bc	52.17 ± 3.74de	19.35 ± 1.85de	7.31 ± 0.53b
Nijisseiki	3,255.42 ± 96.41bc	250.15 ± 40.99a	345.83 ± 29.29a–d	1,964.83 ± 122.85c	697.09 ± 54.3bcd	7.68 ± 1.18a	10.62 ± 0.86bc	60.33 ± 2.35b	21.46 ± 2.26cd	7.96 ± 1.03b

**Notes.**

Data are presented as the mean ± standard deviation (SD) of three biological replicates. Different letters indicate significant differences among cultivars based on one-way ANOVA followed by Duncan’s multiple range test (*p* < 0.05).

The PCA was then used to screen out the main indices for distinguishing high and low cold-resistant germplasms. By integrating the results of the correlation analysis and PCA, four physiological indices (REC, BW/FW, SS, and MDA) and four branch structural indices (XR, X/C, CR, and PR1) were identified as essential criteria for evaluating pear cold resistance.

### Analysis of the mean membership function

The mean values of membership functions were calculated based on four physiological and biochemical indices and four anatomical structure indices. The membership function analysis demonstrated that ‘Hanxiang’ exhibited the highest average membership value (0.90) under the current indicator system and evaluation framework (Table 4). The cold resistance of 12 pear varieties were divided into three grades according to membership function values: ‘Nanguoli’, ‘Hanxiang’, ‘Huagai’, and ‘Zaosu’ were classified as HR type, with mean membership function values ranging from 0.80 to 0.90; ‘Wanyu’, ‘Shuoyu’, ‘Lvbaoshi’, and ‘Hongxiangsu’ were classified as MR type, with mean membership function values between 0.41 to 0.55; and ‘Butirra Rosata Morettini’, ‘Hosui’, ‘Red Doyenne du Comice’, and ‘Nijisseiki’ were identified as LR type, with scores ranging from 0.12 to 0.30. The membership-function-based classification was consistent with the LT50-based cluster classification.

**Table 4:** Membership function values of 12 pear varieties under low–temperature stress.

**Variety**	**LT**_**50**_	**REC**	**CR**	**PR1**	**XR**	**X/C**	**SS**	**MDA**	**FW/BW**	**Mean** **membership** **function value**	**Cold** **resistance** **type**
Nanguoli	0.89	0.95	0.64	1.00	0.96	0.79	0.79	0.74	1.00	0.86	HR
Huagai	0.89	0.96	0.76	0.96	0.96	0.93	0.37	0.55	0.78	0.80	HR
Hanxiang	1.00	1.00	0.81	0.97	1.00	1.00	0.61	1.00	0.74	0.90	HR
Zaosu	0.88	0.92	0.67	0.73	0.99	0.84	1.00	0.63	0.74	0.82	HR
Wanyu	0.54	0.62	0.20	0.59	0.42	0.19	0.10	0.62	0.57	0.43	MR
Shuoyu	0.42	0.71	1.00	0.34	0.36	0.85	0.58	0.37	0.31	0.55	MR
Lvbaoshi	0.49	0.68	0.23	0.69	0.19	0.11	0.32	0.65	0.35	0.41	MR
Hongxiangsu	0.37	0.89	0.23	0.46	0.20	0.10	0.43	0.79	0.23	0.41	MR
Hosui	0.19	0.38	0.00	0.34	0.23	0.00	0.00	0.58	0.14	0.21	LR
Butirra Rosata Morettini	0.00	0.42	0.30	0.00	0.00	0.06	0.30	0.00	0.00	0.12	LR
Red Doyenne du Comice	0.14	0.04	0.28	0.76	0.17	0.12	0.37	0.32	0.06	0.25	LR
Nijisseiki	0.19	0.00	0.11	0.71	0.64	0.24	0.26	0.41	0.10	0.30	LR

## Discussion

To ensure an accurate evaluation of cold resistance, pear shoots used in this study were collected after natural low-temperature acclimation during the overwintering period. As shown in Fig. 1, multiple sub-zero temperature events occurred at the experimental site during winter, particularly from December to January, when the daily minimum temperature frequently dropped below −15 °C, with extreme lows approaching −20 °C. Such prolonged and repeated low-temperature exposure is essential for inducing cold acclimation in woody plants, leading to physiological and biochemical adjustments that enhance freezing tolerance.

Plant cold resilience is a complicated response mechanism affected by several variables, including physiological, biochemical, and structural factors (Strimbeck et al., 2015; Wanjiku & Bohne, 2015). Among these, LT_50_ is a widely used indicator that reflects a plant’s capacity to withstand cold stress (Kwon et al., 2022; Saadati et al., 2019). This study identified the cold resistance of 122 pear germplasms using the electrical conductivity method. Based on LT_50_ values, these germplasms were then clustered into three cold resistance grades: HR, MR, and LR (Fig. 4). To investigate the physiological and biochemical responses underlying cold stress in pear and establish an integrated trait-based evaluation framework for cold resistance across pear germplasm resources, four representative germplasms from each category were selected to study the differences in cell membrane stability, osmotic regulation ability, antioxidant ability, and anatomical structure of these germplasms under low-temperature stress.

As an important protective barrier for plants, the cell membrane can stand against adversity. Its stability is closely linked to plant cold resistance (Ding, Shi & Yang, 2019). Exposure to low temperatures can enhance cell membrane permeability, resulting in elevated electrolyte leakage, which causes an increase in REC (Shi et al., 2023). In this study, as temperatures declined, the REC showed an upward trend, creating an “S”-shaped curve (Fig. 5A), consistent with the results of previous studies (Reynolds, Lixin & De Savigny, 2014). However, the rising rate of REC varied among germplasms with different cold resistance levels. MR and HR groups showed a rapid increase between −25 °C and −35 °C, while LR germplasms showed a sharp increase at −20 °C. At an extremely low temperature (−40 °C), LR germplasms had the highest REC, whereas HR germplasms had the lowest REC. The differences in REC response to low-temperature stress among germplasms with different cold resistance levels reflect variations in the sensitivity of their cell membranes to low-temperature stress: LR germplasms are more sensitive to low temperatures, showing significant electrolyte leakage (as indicated by increased REC) at relatively higher low temperatures.

MDA accumulation levels under stress reflects the degree of damage to plants (Chen, 1991). In the present study, MDA levels in one-year-old pear shoots showed an overall increasing trend with decreasing temperature (Fig. 5B). However, distinct accumulation patterns were observed among germplasms with different levels of cold resistance. Within the temperature range of 4 °C to −25 °C, MDA content in HR and MR germplasms remained relatively stable. In contrast, MDA levels in LR germplasms increased more rapidly over the same temperature interval and reached peak values at relatively higher stress temperatures (between −20 °C and −25 °C). This pattern was generally consistent with the response of REC. Overall, the results indicate that changes in MDA and REC levels are correlated with differences in cold resistance categories among germplasms, with cold-sensitive germplasms tending to exhibit more pronounced changes in membrane system-related indicators (REC and MDA) at relatively higher stress temperatures. These observations are broadly consistent with previous findings reported by Wang et al. (2018). A plant’s resistance to stress relies significantly on osmotic regulators. Through the accumulation of osmotic adjustment substances, including Pro, SS, and SP, plants are able to modify their cellular osmotic equilibrium to endure stress (Li et al., 2018; Zhang, Yang & Li, 2015). Pro can maintain protein stability and membrane integrity during low-temperature stress, in addition to controlling osmotic balance and clearing ROS (Niu et al., 2023; Soloklui, Ershadi & Fallahi, 2012). In the present study, Pro content in HR and MR germplasms exhibited an overall increase, with Pro levels in most HR and MR germplasms peaking later (peaking at −40 °C) than in LR germplasms (Fig. 6). In contrast, the Pro content in LR germplasms showed an earlier peak (at −20 °C or −25 °C) followed by a decline (Fig. 6). This differing accumulation pattern—delayed peak in HR/MR *versus* an early peak in LR—was consistent with the trend of Pro accumulation seen in pomegranate germplasms (Soloklui, Ershadi & Fallahi, 2012). Due to the hydrophilicity of SP and the ability of SS to regulate cell concentration, fluctuations in SP and SS content can affect cellular osmotic potential, consequently determining freezing tolerance in pear germplasms (Seydel et al., 2022). This study showed that the SP and SS of HR germplasms reached their peak values at lower temperatures and increased more than the SP and SS of LR germplasms (Fig. 7), aligning with the findings of cold resistance studies on *Ziziphus jujube* (Li et al., 2024). This might be because plants accumulate SP and SS to enhance their water-holding capacity when they are under stress from low temperatures, thereby reducing their intracellular freezing point and stabilizing membrane integrity. A correlation analysis showed that SP and SS were significantly negatively correlated and highly significantly negatively correlated with LT_50_ (Fig. 9), respectively (Keunen et al., 2013), indicating that osmotic regulator-related traits are closely associated with cold resistance variation among pear germplasms. Low-temperature stress can lead to elevated levels of ROS, and antioxidant enzymes such as SOD and POD are widely reported to be involved in plant responses to oxidative stress under unfavorable conditions (Xu et al., 2023). To enhance the plant’s cold resistance, they can scavenge ROS, maintain the stability of cell membranes, and regulate normal plant activity (Shi et al., 2023). One previous study found that the SOD activity in walnut branches exhibited a complex pattern of increase, decrease, then increase, and POD activity showed an initial increase, followed by a decrease (Ni, Hu & Xu, 2025). The prickly ash germplasms’ SOD activity first rose and then fell with decreases in temperature, whereas POD activity first fell and then rose (Shi et al., 2023). The SOD activity in pomegranate increased as temperature decreased, but declined below a specific critical temperature threshold (Liu et al., 2018). Evidently, different species exhibit distinct modifications in their enzyme activity under low-temperature induction. In the present study, SOD and POD fluctuated with low temperature stress but showed an overall upward trend (Fig. 8). SOD activity in most of the HR and MR germplasms reached a maximum at −40 °C, which was lower than in LR germplasms. POD was similar to SOD, showing fluctuating changes (Fig. 8). The increasing phase may reflect enhanced antioxidant enzyme activity and its participation in free radical scavenging processes, while under more severe stress conditions, the rapid accumulation of reactive oxygen species (ROS) may constrain further increases in antioxidant enzyme activity (Shi et al., 2023). A correlation analysis showed no significant relationship between SOD or POD activity and LT_50_ (Fig. 9), which may be related to differences in the duration of antioxidant enzyme responses among germplasms. Therefore, relying on a single physiological indicator may be insufficient to accurately assess cold resistance in pear.

The water content and the bound/free water ratio are often associated with metabolic activity and growth dynamics, and have been discussed as factors related to cold resistance in woody tissues. BW, with a freezing point ranging from −25 °C to −20 °C, is less prone to freezing, whereas FW, which can move unrestrictedly, freezes below 0 °C (Wang et al., 2022). Studies have demonstrated that the BW content and BW/FW ratio in branches correlate with cold resistance and can be used as major indicators for evaluating cold resistance (Wu, 2011). In the case of pear branches during dormancy, variations in the BW/FW ratio were observed among different grades of cold resistance (Table 2). HR demonstrated the highest ratio, followed by MR, then LR. Furthermore, BW/FW was negatively correlated with LT_50_, indicating that a higher BW/FW ratio is often associated with a lower freezing temperature threshold. These results are consistent with those observed in grapevines (Wang et al., 2022).

The physiological and metabolic processes of plants, together with the anatomical characteristics of plant branches, have been widely reported to be associated with cold resistance (Xu et al., 2023). In the present study, anatomical structures differed significantly among cold resistance groups. To minimize the influence of absolute branch size, anatomical traits were expressed as relative proportional indices. Xylem cells can withstand low temperatures thanks to their rigid, thick cell walls. This study revealed a significantly negative correlation between XR and LT_50_ (Fig. 9). XR levels were much higher in HR germplasms than in MR and LR germplasms (Table 3), aligning with previous findings in apple (Xu et al., 2023) and peach (Niu et al., 2025) regarding cold resistance. This may be due to the carbohydrates that are transported to stems under low-temperature stress, which could increase sorbitol levels in xylem tissues and may be associated with differences in cold resistance (Ito et al., 2013). Furthermore, the xylem vessels are crucial for water transfer. Previous studies have suggested that xylem-related structural traits may contribute to stress adaptation through effects on tissue rigidity and water transport (Chang et al., 2023; García-Cervigón et al., 2020). The branch cortex primarily consists of living cells that are generally more sensitive to low-temperature stress. Under extreme cold conditions, cortical tissues may undergo structural alterations that are associated with differences in cold resistance. In the present study, HR germplasms had a lower CR value compared to MR and LR germplasms, and the X/C ratio in HR germplasms was significantly higher than that in the other groups (Table 3). These results demonstrate a correlation between cold resistance and these traits (Chang et al., 2023; Ito et al., 2013). PR1 was lower in HR germplasms than in the other groups. This pattern is similar to that reported in walnut (Ni, Hu & Xu, 2025) but inconsistent with those of apple, which may be due to differences between species (Xu et al., 2023).

The intricate physiological process of cold resistance is impacted by multiple factors, such as environment, genetics, and physiology (Niu et al., 2025). In this study, a correlation analysis, PCA, and membership function analysis were combined to thoroughly analyze the cold resistance of pears. These statistical approaches provide a multidimensional and integrated perspective, yet the composite scores and rankings should be interpreted as indicator-based, model-dependent estimates of cold resistance under the experimental conditions. The correlation analysis demonstrated a strong association between LT_50_ and physio-biochemical indicators and the anatomical structure of the branches (Fig. 9). There was also an interaction between anatomical structure and physiological indexes, in which the osmotic adjustment substances, such as SS and SP were both significantly correlated with the anatomical structure; however, the antioxidant enzyme SOD was not related to the anatomical structure of each parameter (Fig. 9). These results indicated that osmoregulatory ability was a crucial determinant of the cold resistance of pear germplasms. The PCA was then used to screen out the main indices for distinguishing high and low cold-resistant germplasms. By integrating the results of the correlation analysis and PCA, four physiological indices (REC, BW/FW, SS, and MDA) and four branch structural indices (XR, X/C, CR, and PR1) were identified as essential criteria for evaluating pear cold resistance (Fig. 10). Additionally, the cold resistance of 12 pear varieties was evaluated using the membership function approach, which improved the reliability of the evaluation. The ranking of the 12 pear germplasms for cold resistance by the membership function approach differed slightly from that of the LT_50_, but their cold resistance grades aligned closely with the LT_50_ cluster analysis (Table 4), thereby affirming the credibility of both methods.

Notably, given known variations in baseline cold resistance among species, species origin may potentially influence the evaluation system employed in this study. This study employed clustering analysis based solely on the lethal temperature (LT_5__0_, Fig. 4). While the resulting groupings correlate with species distribution, they are fundamentally driven by phenotypic traits. Notably, each cold-tolerance group (HR, MR, LR) contained accessions from more than one species, indicating that cold resistance is a continuous trait exhibiting substantial variation both within and across species. The strong correlations identified between LT_50_ and traits like BW/FW and X/C ratio held across this phenotypically classified, mixed-species cohort.

### Limitations and future validation

Although the selected materials encompass the core LT_50_ ranges across different cold-tolerance levels, they do not fully capture the broader spectrum of trait variation within each category. Consequently, the rankings and response patterns identified in this study should be interpreted as model-based, relative evaluations derived from a representative subset, rather than as definitive causal or mechanistic hierarchies of cold resistance across the entire germplasm collection.

In addition, while PCA and membership function–based integration provides an objective approach for summarizing multi-trait variation, the resulting composite scores remain dependent on the selected indicator set and weighting structure.

Accordingly, the primary objective of this study was to establish a preliminary, integrative evaluation framework that explicitly accounts for differences among cold-tolerance levels, rather than to provide a final classification applicable to all pear germplasm resources. The general applicability and robustness of the proposed system require further validation through sensitivity analyses (alternative indicator weighting schemes) and independent biological evidence, including multi-year field observations of freezing injury, overwintering survival, recovery growth, and performance traits across environments. Such efforts will be essential to strengthening the biological interpretation and practical utility of the cold resistance evaluation system.

The key indicators identified in this study serve as stable and reliable phenotypic markers for cold resistance, demonstrating predictive value across diverse genetic backgrounds. However, we acknowledge that the disproportionate representation of certain species within specific cold resistance groups suggests our model may partially reflect species-specific traits. Future validation of these markers within single-species populations or breeding populations is warranted to confirm their universal predictive capability.

## Conclusions

In this study, 122 pear germplasms were classified into high (HR), medium (MR), and low (LR) cold-tolerance categories based on the LT_50_of their one-year branches. A total of 27 pear germplasms were classified as LR, 71 pear germplasms were classified as MR, and 24 pear germplasms were classified as HR. After classification, 12 representative germplasm accessions from different cold-resistance types (HR, MR, and LR) were selected for multi-indicator evaluation. The results indicated that pear germplasms with different cold-resistant types exhibited significant differences in physio-biochemical traits. Compared with LR germplasms, HR and MR germplasms displayed more favorable response patterns in membrane-related and osmotic adjustment-related indicators. In addition, branch anatomical traits were associated with cold resistance categories, with HR germplasms generally showing higher XR and X/C values and lower CR and PR1 values than MR and LR germplasms. Through correlation analysis and PCA, four physiological indices (REC, BW/FW, SS, and MDA) and four branch structural indices (XR, X/C, CR, and PR1) were identified as the primary evaluation criteria for cold resistance in pear germplasms. Additionally, the membership function approach indicated that ‘Hanxiang’ had the greatest mean membership function value (0.90). The rankings provided here are informative for germplasm screening under the current indicator system and evaluation framework but should be validated with independent field performance data before conclusive recommendations for breeding or cultivation can be made. This study established a evaluation system for pear cold resistance, providing a theoretical basis for the future screening of cold resistance pear germplasms.

## Supplemental Information

10.7717/peerj.21475/supp-1 Supplemental Information 1 Characteristics of pear germplasm resources for evaluation Note: Different letters represent different pear species. I represents Interspecific hybridization (accessions derived from parents belonging to different *Pyrus* species were recorded as interspecific hybrids ), P represents *P. pyrifolia* , U represents *P. ussuriensis*, B represents *P. breshneideri*, C represents *P. commusnis*, W represents Wild species

10.7717/peerj.21475/supp-2 Supplemental Information 2 Comparison of low-temperature half-lethal temperatures (LT_50_) among pear germplasm of different cold tolerance levels Note: HR, MR and LR indicate high-, medium- and low-cold tolerance groups.

## Additional Information and Declarations

### Data Availability

The following information was supplied regarding data availability: The data is available in the Supplemental Files. The data is available at Zenodo: liu186253. (2025). liu186253/Data: raw data (Version V11). Zenodo. https://doi.org/10.5281/zenodo.17524773.
