# Supplementary material for: Evaluation of cold resistance in pear (Pyrus L.) germplasms: integrating physiological and biochemical responses with anatomical traits under low temperature stress
Source: PeerJ. 2026 Jun 29;14:e21475. doi: 10.7717/peerj.21475 (PMC13326648; doi:10.7717/peerj.21475)
Supplement: Supplemental Information 1 — Note: Different letters represent different pear species. I represents Interspecific hybridization (accessions derived from parents belonging to different Pyrus species were recorded as interspecific hybrids ), P represents P. pyrifolia , U represents P. ussuriensis, B represents P. breshneideri, C represents P. commusnis, W represents Wild species [file peerj-14-21475-s001.docx]

**Supplementary Data**

**Table S1** Characteristics of pear germplasm resources for evaluation

| **No.** | **Germplasm** | **Species** | **No.** | **Germplasm** | **Species** |
| --- | --- | --- | --- | --- | --- |
| P1 | Shanli | U | P35 | Lvbaoshi | I |
| P2 | Hanhong | I | P36 | 09-01-110 | B |
| P3 | Hanxiang | I | P37 | Yaguangli | U |
| P4 | Hebeiduli | W | P38 | Zaosumi | I |
| P5 | Nanguoli | U | P39 | Jieshishan wild pear | W |
| P6 | Hansu | I | P40 | Qiuyue | B |
| P7 | Xiaohuagai | U | P41 | Jiyu | C |
| P8 | Zaosu | I | P42 | Yali | B |
| P9 | Qiyuesu | I | P43 | Jinzhui | B |
| P10 | Hanlu | I | P44 | Zeibutou | W |
| P11 | Ganlizao8 | I | P45 | 11-11-141 | I |
| P12 | Pingguoli | P | P46 | Boli | B |
| P13 | Zheli | I | P47 | Honghuaguan | U |
| P14 | Hongyueli | I | P48 | Shuipingxiao | B |
| P15 | Huanghuaguan | U | P49 | Xinli No.7 | I |
| P16 | Waibali | U | P50 | Xiangchunli | B |
| P17 | Pingboxiang | I | P51 | Shuoyu | I |
| P18 | Hongpisuanli | U | P52 | Manyu | I |
| P19 | Qiubaili | B | P53 | 11-04-139 | B |
| P20 | Guali | U | P54 | Banjinsu | B |
| P21 | Gualizi | U | P55 | Wild pear | W |
| P22 | Fojianxi | B | P56 | Zaoshu | U |
| P23 | Pingdingxiang | B | P57 | Whangkeumbae | P |
| P24 | Zaosuhong | I | P58 | Wujiuxiang | I |
| P25 | Mili | B | P59 | Nanhong | U |
| P26 | Sucui No.1 | P | P60 | Zunhuazaobai | B |
| P27 | Miyu | I | P61 | Canyu | C |
| P28 | 03-21-15 | I | P62 | Xueying | I |
| P29 | Wanyu | B | P63 | Douli | W |
| P30 | Suyu | I | P64 | Jingbaili | U |
| P31 | 11-02-257 | B | P65 | 8-42 | C |
| P32 | 03-19-99 | B | P66 | Xiangsuli | P |
| P33 | 10-24-20 | B | P67 | Xuehuali | B |
| P34 | Zaoxiangli | U | P68 | Xueqing | I |

**Table S1 (continued)** Characteristics of pear germplasm resources for evaluation

| **No.** | **Germplasm** | **Species** | **No.** | **Germplasm** | **Species** |
| --- | --- | --- | --- | --- | --- |
| P69 | Hongzhimuyang | B | P96 | Hosui | P |
| P70 | Weiningdahuang  ahuang | P | P97 | Nijisseiki | P |
| P71 | 8-26 | I | P98 | Manaoli | I |
| P72 | Hongxiao | B | P99 | Aiganshui | P |
| P73 | Hongxiangsu | I | P100 | Red Doyenné du Comice | C |
| P74 | Xuefeng | I | P101 | New Early Barlett | C |
| P75 | Pingguhongli | P | P102 | Clapp’s Liebling | C |
| P76 | Xiangli | P | P103 | Alexandrine Douillard | C |
| P77 | Xianghong | C | P104 | Santa Maria Morettini | C |
| P78 | Huangguan | I | P105 | Xinchengyangli | C |
| P79 | Manfeng | P | P106 | Packham’ Triumph | C |
| P80 | Shuihongxiao | P | P107 | Le Lectier | C |
| P81 | Qiyuehongxiangli | B | P108 | Avicadi | C |
| P82 | Hongli | P | P109 | Red d’ Anjou | C |
| P83 | Hongxing | C | P110 | Conference | C |
| P84 | Xianghongmi | I | P111 | Beurré Giffard | C |
| P85 | Yangbai | I | P112 | Butirra Rosata Morettini | C |
| P86 | Hongtangli | P | P113 | Xianmei | C |
| P87 | Jinxiangmi | P | P114 | Gute Luise | C |
| P88 | Bartlett | C | P115 | Hardy | C |
| P89 | Yuluxiang | I | P116 | Coscia | C |
| P90 | Yumeiren | B | P117 | Decola | C |
| P91 | Shijiwuhe | B | P118 | Boliarska | C |
| P92 | Cibaili | C | P119 | Abbé Fétel | C |
| P93 | Mansoo | P | P120 | Itali No.2 | C |
| P94 | Cuiyu | I | P121 | Yubileen Dar | C |
| P95 | Baozhuli | P | P122 | Jana | C |

Note: Different letters represent different pear species. I represents Interspecific hybridization (accessions derived from parents belonging to different *Pyrus* species were recorded as interspecific hybrids ), P represents *P. pyrifolia*, U represents *P. ussuriensis*, B represents *P. breshneideri*, C represents *P. commusnis*, W represents Wild species
