# Supplementary material for: Evaluation of cold resistance in pear (Pyrus L.) germplasms: integrating physiological and biochemical responses with anatomical traits under low temperature stress
Source: PeerJ. 2026 Jun 29;14:e21475. doi: 10.7717/peerj.21475 (PMC13326648; doi:10.7717/peerj.21475)
Supplement: Supplemental Information 2 — Note: HR, MR and LR indicate high-, medium- and low-cold tolerance groups. [file peerj-14-21475-s002.docx]

**Supplementary Data**

**Table S2** Comparison of low-temperature half-lethal temperatures (LT_50_) among

pear germplasm of different cold tolerance levels

| Cold tolerance type | Low-temperature half-lethal temperatures (LT_50_) | | | | |
| --- | --- | --- | --- | --- | --- |
|  | Min（℃） | Max （℃） | Mean（℃） | Standard  difference | Coefffcient of  variation (%) |
| HR | -35.68 | -32.17 | -33.67 | 0.85 | 2.54% |
| MR | -31.42 | -28.65 | -29.97 | 0.75 | 2.52% |
| LR | -28.24 | -25.98 | -26.94 | 0.68 | 2.51% |

Note: HR, MR and LR indicate high-, medium- and low-cold tolerance groups.
